# Behavioral Assessment and Treatment via Telehealth for Children with Autism: From Local to Global Clinical Applications

**DOI:** 10.3390/ijerph19042190

**Published:** 2022-02-15

**Authors:** Kelly M. Schieltz, Matthew J. O’Brien, Loukia Tsami, Nathan A. Call, Dorothea C. Lerman

**Affiliations:** 1 Stead Family Department of Pediatrics, The University of Iowa Stead Family Children’s Hospital, Iowa City, IA 52242, USA; matthew-j-obrien@uiowa.edu; 2 Center for Autism and Developmental Disabilities, University of Houston, Clear Lake, Houston, TX 77004, USA; tsami@uhcl.edu (L.T.); lerman@uhcl.edu (D.C.L.); 3 Department of Pediatrics, Children’s Healthcare of Atlanta and Emory University, Atlanta, GA 30329, USA; nathan.call@choa.org

**Keywords:** telehealth, applied behavior analysis, functional analysis, functional communication training, challenging behavior

## Abstract

Functional analyses (FA) and functional communication training (FCT) are the most commonly used behavioral assessment and treatment approaches via telehealth for children with autism spectrum disorder (ASD) who display challenging behavior. The FA + FCT telehealth model has been shown to maintain treatment effectiveness (i.e., child behavioral outcomes and parent acceptability), as well as demonstrate treatment efficiency (i.e., cost savings). However, the majority of these studies have been conducted in the United States. Therefore, the purpose of this study was to evaluate the outcomes obtained with the telehealth FA + FCT model that included global applications. Descriptive statistics were used to analyze the results of the 199 participants who enrolled in the telehealth project across all project sites. The results showed that behavioral outcomes and parent acceptability maintained at similar levels to previous studies across all sites. Additionally, very few differences were found across project sites in relation to drop-out rates, visit cancellations, and technology issues. These results demonstrate the effectiveness of the FA + FCT telehealth model for addressing the challenging behavior needs of children with ASD globally and highlight areas in need of additional evaluation (e.g., drop-outs, cancellations) to determine the conditions under which telehealth could be best used.

## 1. Introduction

Applied behavior analysis (ABA) uses the principles of behavioral science to understand how behavior is affected by the environment (behavioral assessment) and how to bring about changes in behavior by increasing positive behavior and decreasing challenging behavior (behavioral treatment) [1]. ABA is considered to be the treatment of choice for reducing challenging behavior, especially for persons with autism spectrum disorder (ASD) [2].

Specific to the behavioral assessment and treatment of challenging behavior, the most widely used ABA assessment and treatment procedures are functional analysis (FA) [3,4] and functional communication training (FCT) [5,6], respectively. The purpose of an FA is to identify the environmental conditions under which challenging behavior occurs, which is then used to develop a treatment plan such as FCT. In FCT, challenging behavior is replaced with a communicative alternative, whereby the communication response results in the reinforcer that was identified in the FA.

This FA + FCT model has been shown to be effective across behaviors, populations, and settings (e.g., [7,8,9,10,11]). For example, Wacker et al. [12] showed, on average, a 90% reduction in challenging behavior from baseline levels following FCT treatment across 84 children with developmental disabilities, who were between the ages of 14 and 83 months old. These results were obtained over an 18-year period, whereby behavioral therapists visited the children’s homes and coached their parents on how and when to implement the assessment and treatment procedures. Although these results showed the effectiveness of the FA + FCT model in treating challenging behavior displayed by young children, the model was resource-intensive, or inefficient, in terms of cost, travel time, and geographical reach. Therefore, the consideration of other service delivery models that maintain the positive treatment effects of FA + FCT but improve its efficiency is warranted. One such delivery model is telehealth.

Telehealth, a model of delivering clinical services synchronously or asynchronously via electronic communication [13], has been utilized within healthcare for decades [14]. The use of this service delivery model fulfills the healthcare “triple aim” of enhancing healthcare experiences, improving population health, and reducing costs [15]. In ABA, the effectiveness and efficiency of telehealth for addressing the behavioral needs of young children with ASD was demonstrated in a study that compared three service delivery models: in vivo in-home, clinic-to-clinic telehealth, and clinic-to-home telehealth [16]. The results of this study showed that (a) the challenging behavior displayed by children with ASD or other developmental disabilities was reduced to similar levels following behavioral treatment, and (b) treatment acceptability as rated by the children’s parents remained at similarly high levels across all three models. There were no significant differences between these two outcome measures across service delivery models, suggesting that the two telehealth models that used the same behavioral assessment and treatment procedures were as effective as the in vivo in-home model. In contrast, significant differences were obtained in terms of cost-of-service delivery between the in vivo in-home model and telehealth models. That is, the cost-of-service delivery was significantly less when using the telehealth models than the in vivo model, which was likely accounted for by the reduction in or elimination of parent and therapist travel expenses. Additionally, the geographical reach was significantly greater with the telehealth models than the in vivo model (i.e., 160 km radius for the in vivo in-home model versus statewide reach for the telehealth models). Taken together, these results showed that the telehealth model of delivering behavioral assessment and treatment improved the efficiency of service delivery.

Although the initial use of telehealth to deliver FA + FCT maintained effectiveness and increased the efficiency of addressing the behavioral needs of young children with ASD, the ABA telehealth literature largely remained in its infancy prior to the COVID-19 pandemic, even though telehealth had been utilized within this specialty since the late 1990s. To guide practitioners early on in the COVID-19 pandemic, Schieltz and Wacker [17] summarized the existing ABA literature focused on behavioral assessment and treatment of challenging behavior. This review found 18 published studies, with approximately 40% of them (more than 50% of the summarized participants) published by our research team at the University of Iowa. Similar to the effectiveness outcomes reported by Lindgren et al. [16], improvements in behavior occurred for the majority of children (98%) and treatment acceptability was high. However, most of the studies targeted children under the age of 6 years who resided within the United States. Only one published study reported results for children with ASD who resided in countries outside the United States (*n* = 13) [18]. With the increased use of telehealth, as prompted by the COVID-19 pandemic to maintain delivery of behavioral services to children with ASD, the effects of the FA + FCT telehealth model are needed across more recent national and global clinical replications to ensure their effectiveness and efficiency, as well as to understand the limits of their use. 

The purpose of this study was to conduct a retrospective descriptive analysis of the national and global clinical applications of the FA + FCT telehealth model. Our primary research questions asked if (a) child and (b) parent outcomes maintained their effectiveness across broader geographical regions within the United States and international countries. Our secondary research questions asked if variables related to discontinuation, cancellation, and technology differed across national and international geographical regions given that these variables have not been evaluated within the ABA telehealth literature to date. To answer these questions, we evaluated child and parent outcomes, as well as the secondary outcome measures across our site in Iowa, two of our partner sites within the United States (Georgia and Texas), and one of our partner sites (Texas) that implemented the procedures with families residing in other countries worldwide. These sites were chosen because they implemented the same behavioral assessment and treatment model (FA + FCT) as Wacker et al. [19,20]. With our goal to demonstrate the continued effectiveness and efficiency of the FA + FCT telehealth model, we hypothesized that child and parent outcomes would be similar across sites and to the results obtained in previous studies. Related to the secondary measures, we hypothesized that outcomes would be similar across sites, but that the international sites may demonstrate more technology-related issues.

## 2. Materials and Methods

### 2.1. Participants

Participants were enrolled in one of two research projects: (1) a federally funded project [21] that was conducted with families residing in three different locations in the United States (Iowa, Georgia, and Texas; henceforth US project) or (2) a university project in Texas that conducted all procedures with families residing in countries outside the United States (henceforth international project). Criteria for inclusion in the US project were that the children (a) were between the ages of 18 months and 6 years, 11 months; (b) were diagnosed with ASD; (c) lived or received services in one of the participating states (Iowa, Georgia, Texas); and (d) displayed challenging behavior as indicated by a score of 13 or greater on the Aberrant Behavior Checklist-Irritability subscale. Criteria for inclusion in the international project were that the children (a) were between the ages of 18 months and 12 years, 11 months; (b) were diagnosed with a developmental disability; and (c) engaged in challenging behavior on a daily basis, as determined by a score of 12 or greater on the Aberrant Behavior Checklist-Irritability subscale. Additionally, the family had to have access to an internet-enabled device (e.g., laptop, computer, tablet, or smartphone) and high-speed internet (minimum download/upload speeds of 400 kbps/400 kbps) either in their home or a community setting. All children who participated in the US and international projects were included in this study (*n* = 199). 

### 2.2. Settings and Materials

#### 2.2.1. Participants

Behavioral therapists for both the US and international projects coached parents of the participating children to conduct all assessment and treatment procedures within rooms (e.g., bedrooms, living rooms; community learning center for one international participant) within their homes. Equipment included hardware (e.g., laptop, tablet, or smartphone, internal or external webcam), software (e.g., videoconferencing applications such as Ring Central^TM^, Vidyo^TM^, Vsee, Webex^TM^, Zoom), and high-speed internet access. For families enrolled in the US project who did not have adequate equipment, the study team loaned equipment via the project’s equipment lending library (i.e., hardware, software) or purchased access for the duration of project enrollment (i.e., internet). For the international project, access to devices and high-speed internet were required to participate in the project. 

Across both the US and international projects, work tasks and leisure items found within the home were used during the relevant assessment and treatment sessions. For the US project, communication options (e.g., picture cards, voice output devices) were provided to families who lacked access to the necessary communication modality. For the international project, communication options (e.g., picture cards) were created by the families with materials available within their homes. 

#### 2.2.2. Therapists

All behavioral therapists provided coaching on the assessment and treatment procedures from office spaces or labs at their respective sites across both the US and international projects. All behavioral therapists utilized Windows-based PCs, large video monitors, external webcams, headsets, teleconferencing software (e.g., Ring Central^TM^, Vidyo^TM^, Vsee, Webex^TM^, Zoom), and video recording software (e.g., Debut^TM^).

#### 2.2.3. Interpreters

The international project was the only project that utilized interpreters. The function of the interpreter was to only translate the spoken words of the behavioral therapist, without adding personal thoughts or other directions. All interpreters were recruited as volunteers (a) during graduate level course discussions on research opportunities available at the university in Texas or (b) through social media posts. Prior to interpreting FA and FCT sessions with the participating family, all interpreters met with the lead behavioral therapist (third author) for a one-hour training on the assessment and treatment protocols. The goal of the training was to familiarize the interpreter with the procedures and rationales, not train them on how to independently conduct procedures, coach the families to conduct procedures, or consult with families on areas of concern. 

All interpreters provided live translation of the behavioral therapist’s coaching from one of three general locations: (1) Texas site where the behavioral therapist was present, (2) family home, or (3) a location separate from the behavioral therapist and participating family (e.g., interpreters’ home or office). At the Texas site, all equipment was the same as described above. At the family’s home and interpreter’s home or office, equipment included personally owned devices such as laptops or computers and software that included the same programs used by the behavioral therapist and family (e.g., Vidyo^TM^, Zoom, Ring Central^TM^).

### 2.3. General Study Procedures

Across both the US and international projects, procedures were conducted in the same manner as described by Wacker et al. [19,20].

#### 2.3.1. Telehealth Visits

Parents were coached by behavioral therapists to conduct FA and FCT procedures during weekly 1 h appointments. Weekly appointments were scheduled at a time convenient to both the therapists and families. For most participants in the US project, appointments were scheduled during business or evening hours of the workweek, with occasional appointments scheduled on weekends. For the international project, weekly appointments considered time zone differences, resulting in the behavioral therapists in Texas frequently conducting appointments (a) early in the morning (e.g., 5:30 AM Texas time, 6:30 PM Vietnam time) or late at night (e.g., 10:30 PM Texas time, 7:30 AM Pakistan time), especially for participants residing in the Middle East and Asia and (b) during weekends when families did not have competing work responsibilities.

#### 2.3.2. Functional Analysis

Behavioral therapists coached parents via telehealth to conduct all FA procedures within a multielement single-case experimental design based on the procedures described by Iwata et al. [4]. All FA sessions were conducted for 5 min, with three to five sessions conducted during each weekly appointment. All FA sessions were videorecorded for scoring at a later point in time, by independent data collectors. Each FA session evaluated one social (i.e., escape, tangible, or attention) or control (i.e., free play) condition (see Iwata et al. [4] and Wacker et al. [19] for more complete descriptions of the FA test and control conditions). During an escape session, the parent instructed the child to complete tasks (e.g., “point to the dog”) intermittently throughout the session. During tangible and attention sessions, the parent restricted the child’s access to preferred leisure activities and parental attention, respectively. Across all sessions, reinforcement (i.e., providing a break from the task, access to the preferred leisure activities, access to parental attention) was provided when challenging behavior occurred. During a free play session, which served as the control condition, the parent provided the child with access to preferred leisure activities and parental attention, while refraining from instructing the child to complete tasks. If challenging behavior occurred in a free play session, it was ignored. FA sessions were continued until stable patterns of challenging behavior were obtained that determined the conditions that functioned to occasion and reinforce the child’s challenging behavior. 

#### 2.3.3. Functional Communication Training

Behavioral therapists coached parents via telehealth to conduct all FCT procedures within a single-case experimental design, with the most common design being a reversal design. All sessions were conducted for 5 min, videorecorded, and scored at a later point in time. Three to five sessions were conducted per appointment. Baseline sessions consisted of extinction (same procedures as the FA except reinforcement was not provided for the occurrence of challenging behavior) or the sessions from the relevant FA condition (i.e., condition identified as functioning to cause and reinforce the child’s challenging behavior). During FCT sessions, the parent presented the child with an opportunity to communicate for the relevant reinforcer following a specified requirement (e.g., completing a task instruction, waiting for a specified amount of time). When there was a communication opportunity, the parent presented the child with a communication stimulus (e.g., a picture card or voice output device) and informed the child that he/she could communicate. If the child did not communicate within 5 s, the parent followed a prompting sequence (i.e., vocal prompt, model prompt, physical prompt) to teach the child that his/her communication would result in the relevant reinforcer. When the child communicated, the parent provided praise and access to the relevant reinforcer for 1–2 min. When challenging behavior occurred, the reinforcer was withheld (i.e., task remained present, access to leisure activities or attention remained restricted). 

FCT treatment plans were developed using the results of the FA and discussions with the child’s parent. For example, if the FA identified escape from tasks as reinforcing the child’s challenging behavior, then the FCT treatment plan focused on the child requesting a break from the task after completing a specified number of task requests. If the FA identified access to tangible items or attention as reinforcing the child’s challenging behavior, then the FCT treatment plan focused on the child requesting access to preferred leisure activities or attention, respectively, following a specified wait period without access to the relevant reinforcer. All FCT treatment plans began with small requirements (e.g., complete one task, wait without access for 5 s) that were gradually increased as the child demonstrated little to no challenging behavior and independent communication.

### 2.4. Dependent Variables and Interobserver Agreement

#### 2.4.1. Challenging Behavior and Independent Communication

The primary dependent variables for children across both the US and international projects were challenging behavior and independent communication. *Challenging behavior* was defined individually for each child and included at least one of the following topographies: aggression, property destruction, self-injury, and tantrums. In general, aggression was defined as challenging behavior directed towards others (e.g., hitting, kicking, head butting, biting), property destruction was defined as challenging behavior directed towards objects (e.g., throwing items, breaking materials), self-injury was defined as challenging behavior directed towards oneself (e.g., head banging, hand biting), and tantrums were defined as vocalizations above a conversational level (e.g., screaming, yelling) as well as other challenging behavior that would not cause harm to self, others, or objects (e.g., crying, flailing). Challenging behavior was measured using frequency (aggression, property destruction, self-injury) or duration (tantrums) scoring methods. Data were collected for each session by trained data collectors using an electronic data collection system (BDataPro) [22]. For challenging behavior scored using a frequency measure, data were converted into responses per minute by dividing the total frequency by 5 min. For challenging behavior scored using a duration measure, data were converted into a percentage of session time by dividing the total duration of challenging behavior by the total session length and multiplying by 100. When targeted challenging behavior included behavior scored using both frequency and duration measures, data were converted into a percentage of 10 s intervals by calculating the number of intervals with which challenging behavior occurred, dividing by 30 intervals and multiplying by 100. 

*Independent communication* was defined as the child requesting the relevant reinforcer without parental prompting. Independent communication was measured using a frequency scoring method, and data were converted into a percentage across communication opportunities. This percentage was calculated by dividing the total number of independent communication responses by the total number of communication opportunities offered per session and multiplying by 100.

#### 2.4.2. Interobserver Agreement

A second trained data collector independently scored the occurrence of challenging behavior and independent communication displayed by the children as a measure of interobserver agreement (IOA). IOA was calculated using a partial interval agreement method. Specifically, each session was divided into 10 s intervals. In each interval, the lower number of behavioral occurrences was divided by the higher number of behavioral occurrences to obtain a ratio between 0 and 1. All ratios were then summed and divided by the total number of intervals, which was then multiplied by 100. Across both the US and international projects, IOA was scored across an average of 51% and 44% of FA and FCT sessions, respectively. IOA averaged 97% (range, 82% to 100%) for the FA and averaged 96% (range, 83% to 100%) for FCT.

### 2.5. Procedural Fidelity and Interobserver Agreement

Parent procedural fidelity for those who completed the project was scored by trained data collectors across both the US and international projects to ensure the parent’s accuracy with implementation of the FA and FCT procedures being coached by the behavioral therapist. For both the FA and FCT, procedural fidelity was measured using the same task analysis scoring systems as Wacker et al. [19] for the FA and Suess et al. [23] for FCT. Procedural fidelity was calculated by dividing the total the number of steps completed accurately by the total number of steps in the task analysis and multiplying them by 100.

A second trained data collector independently scored FA and FCT sessions for parent procedural fidelity to obtain a measure of IOA. IOA was scored using a total agreement measure, whereby each procedural fidelity step in the task analysis was compared. If both observers scored the step as occurring, an agreement was scored. If one observer scored a procedural fidelity step as not occurring and one scored the step as occurring, a disagreement was scored. IOA was then calculated by dividing the total number of agreements by the total number of agreements plus disagreements and multiplying by 100. Of the total sessions scored for procedural fidelity, IOA was scored across an average of 65% and 62% of FA and FCT sessions, respectively, across the US and international projects. IOA averaged 97% (range, 44% to 100%) in the FA and averaged 96% (range, 89% to 100%) in FCT.

### 2.6. Data Analysis

Descriptive statistics (mean, range, standard deviation, range) were used to summarize the outcomes of the FA + FCT telehealth model across the US and international projects. Data comparisons were conducted across project sites with the US sites consisting of data from Iowa, Georgia and Texas separately, and the international site consisting of data from countries outside the United States.

#### 2.6.1. Project Status Outcomes

All participants were categorized as *completers* or *non-completers* according to the project phase in which they discontinued participation. Completers were defined as participants who completed the FA and FCT phases. Non-completers were defined as participants who discontinued participation prior to the completion of FCT.

#### 2.6.2. Outcomes for Completers

Outcome data were compared across project sites for (a) behavioral functions identified in the FA and targeted in FCT, (b) percent reduction in challenging behavior at the completion of FCT, (c) treatment acceptability ratings at pre- and post-treatment timepoints, and (d) parent procedural fidelity.

*Behavioral function(s)* was determined at the completion of the FA for all participants (completers and non-completers) who completed a FA (*n* = 146). To identify the function(s) maintaining each child’s challenging behavior, visual inspection criteria were used as outlined by Roane et al. [24]. FA outcomes were categorized as the functions of (a) escape, (b) tangible, (c) attention, (d) automatic, and (e) no function identified. Except for the category of no function identified, more than one function could have been identified as maintaining the child’s challenging behavior. Frequencies in each functional outcome category were summed, divided by the total number of FAs completed across participants, and multiplied by 100. *Targeted function(s) in FCT* were measured in the same manner used for behavioral function, except that the frequencies summed were from all participants (completers and non-completers) who began FCT (*n* = 129). 

*Percent reduction of challenging behavior* was the primary outcome measure for both the US and international projects. Percent reduction was calculated for each participant by averaging the occurrence of challenging behavior during the initial baseline and the occurrence of challenging behavior during the final three FCT treatment sessions. The average occurrence during the final three treatment sessions was divided by the average occurrence during baseline and multiplied by 100. This result was then subtracted from 100. The percent reduction outcome was used to determine treatment success, which was defined as reductions in challenging behavior of 80% or greater. Percent reduction outcomes for each participant who completed FCT (completers; *n* = 97) were averaged for comparisons across sites. 

*Treatment acceptability* was measured after the first and last FCT treatment appointments, whereby parents rated, using a 7-point Likert scale, the first item on the Treatment Acceptability Rating Form-Revised (TARF-R) [25]. This item measured their acceptability of the FCT treatment, with options ranging from *not at all acceptable* (1) to *very acceptable* (7). This item specifically asked, “How acceptable do you find the treatment to be regarding your concerns about your child?”. Across participants who completed FCT (*n* = 97), TARF-R ratings were averaged at pre- and post-treatment measurement time points. 

*Procedural fidelity* was measured using the procedures described above. Fidelity was evaluated by averaging the obtained outcomes of the FA and FCT across participants who completed FCT (*n* = 97).

#### 2.6.3. Outcomes for Non-Completers

Outcome data for non-completers were compared across project sites for (a) project phases which they discontinued and (b) number of weeks enrolled in the project. *Project phase* discontinuation was classified into the following categories: prior to the FA, during the FA, prior to FCT, and during FCT. For each discontinuation category, the number of participants who discontinued within that phase was summed, divided by the total number of participants who discontinued participation across all categories, and multiplied by 100. Within these categories, *weeks enrolled* was calculated by counting the number of elapsed weeks from the child’s enrollment week (date of consent) to their discontinuation week (date of discontinuation). Across participants, the number of weeks enrolled was averaged within each discontinuation category.

#### 2.6.4. Outcomes for Completers and Non-Completers

Outcome data were compared across project sites and completion status for (a) visit cancellations and (b) technology issues. Across weekly appointments both the US and international projects measured cancellations and technology issues. *Cancellations* were defined as weekly visits not completed because of a priori cancellations by the parent or therapist, as well as parent failures to show up to the weekly visit. Cancellations were calculated by dividing the number of cancellation weeks by the total number of enrolled assessment and treatment weeks and multiplying by 100. *Technology issues* were calculated by dividing the number of documented technology issues (e.g., audio or video feed issues) by the total number of enrolled assessment and treatment weeks minus the number of cancelled weeks. This total was then multiplied by 100.

## 3. Results

### 3.1. Participants

#### 3.1.1. Child Participants

Table 1 lists characteristics of all of the enrolled participants across the US and international projects. Across the 199 children enrolled in the US and international projects, children’s ages averaged 56.4 months, and the majority of children were male (79.9%). Only the age of children from the international project participants differed across sites. Across sites, age ranged from an average of 49.9 to 71.5 months of age and the proportion of males ranged from 75.7% to 83.9%. Except for two children in the international project, all children had a diagnosis of ASD (99% across all sites). For the US project, the majority of children were White (83.1% Iowa, 59.5% Georgia, 69.6% Texas). For the international project, children were from 21 different countries across five continents, with the majority residing in Greece (17.0%) and Pakistan (12.8%). Across all projects, 19.1% of children were Hispanic or Latino. Specifically, in the US project, 44.6%, 8.5%, and 5.4% of children were Hispanic or Latino across the Texas, Iowa, and Georgia sites, respectively. In the international project, 12.8% of children were Hispanic or Latino. The one-way distance from the behavioral therapist to the children ranged from 4.8 km to 13,928.9 km. On average, the one-way distance was shortest for children residing in Texas (*M* = 57.5 km) and farthest for children residing in international countries (*M* = 10078.5 km). Across both US and international projects, mothers (93%) most frequently implemented FA and FCT procedures with coaching from a behavior therapist. In the international project, interpreters were used for 57.4% of children.

#### 3.1.2. Behavior Therapists

Table 2 lists characteristics of the behavior therapists across the US and international projects. Behavior therapists ranged in age from 24 to 53 years old (*M* = 38.3 years). Most behavior therapists were female (80%) and White (60%). None of the behavior therapists were Hispanic or Latino. For the international project, the countries of origin of the three behavior therapists were Greece, India, and Turkey. The education level of the behavior therapists ranged from bachelor’s degrees (20%) to doctoral degrees (50%). Additionally, 50% of behavior therapists held behavior analytic board certification at master’s level (BCBA, 30%) or doctoral level (BCBA-D, 20%), and 40% were licensed psychologists. The behavior therapists had an average of 114 months of experience in working with individuals with ASD (range, 12–228 months), 143.2 months of experience in behavior analysis (range, 24–408 months), 126.1 months of experience conducting FAs and FCT (range, 3–396 months), and 20 months of telehealth experience (range, 0–80 months). 

#### 3.1.3. Interpreters

Table 3 lists the characteristics of the interpreters used in the international project. Eleven interpreters between the ages of 24 and 42 years old (*M* = 31.6 years) provided interpreter services for 25 (57.4%) different international families. Interpreters were mostly female (91%), and non-Hispanic or Latino (81.8%). Most were current graduate students (63.6%) when they provided interpretations for the international project. Each interpreter came from a different country of origin, which included five different continents. Interpreters’ experiences within the family’s country ranged between 0 and 33 years (*M* = 15.9 years; SD = 10.0). Families requesting/requiring interpreter services came from 15 different countries with the most represented countries being Mexico, Morocco, Saudi Arabia, and Vietnam. Nine different languages were spoken with the most common language for interpretation being Arabic. The next most common languages used for interpretation were Spanish and Vietnamese, followed by French and Russian. Other languages included Farsi, Mandarin, Nepalese, and Urdu. Most interpreters did not have prior experience with FA and FCT (91%). When providing interpretation services, interpreters were most often located in the United States but in a separate location from the behavioral therapist who provided coaching on FA and FCT procedures (44%). Other locations of the interpreter included the same room as the behavioral therapist providing coaching (28%), in a country separate from the family and behavioral therapist (20%), and in the family’s home (12%). 

### 3.2. Outcomes

**Figure 1:**
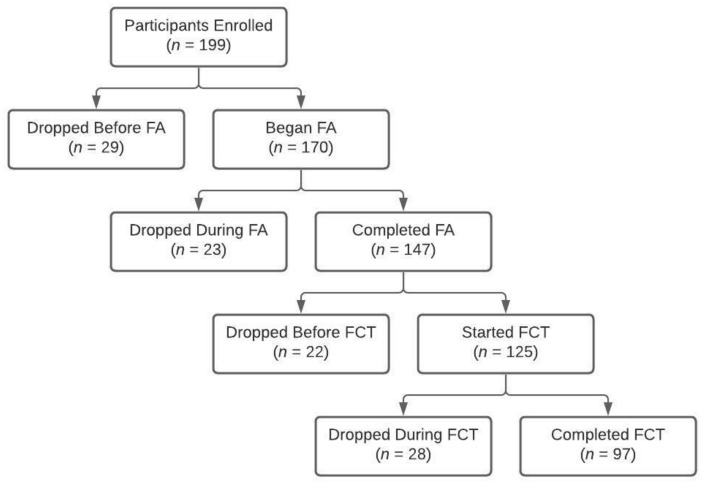
Project status outcomes of discontinuation and completion by study phase.

Figure 1 displays the project status outcomes for all participants across the US and international projects. Across all projects, 199 participants consented and were subsequently enrolled in their respective project. Twenty-nine participants discontinued participation from the project before beginning the FA and another 23 participants discontinued participation from the project during the FA, resulting in 147 participants completing the FA. Of the 147 participants who completed the FA, 22 participants discontinued participation in the project before FCT was initiated and another 28 participants discontinued participation during FCT, resulting in a total of 97 participants (49%) completing the project across all sites.

#### 3.2.1. Outcomes for Completers

Of the participants who started and completed the FA (*n* = 147), individual results were visually inspected to determine the behavioral function(s) maintaining the child’s problem behavior (Table 4). Across all sites, tangible (66.7%) and escape (56.5%) functions were identified most often as maintaining the participants’ challenging behavior. Attention functions were identified in 25.9% of participants, and automatic functions were identified in 0.7% of participants. In 12.9% of participants, a behavioral function for challenging behavior could not be identified because of the absence of challenging behavior occurring during the FA. When aggregated by project site, similar outcomes were observed. That is, escape and tangible functions were the most frequently identified functions, and automatic functions were rarely identified; however, FA conditions used to evaluate for an automatic function (i.e., alone) were not conducted because we do not know how best to conduct that condition within families’ homes when supervision cannot be provided in person. Relative to the identification of an attention function, the Texas site had the most participants consistently display challenging behavior in this condition (50%). In contrast, the Iowa site had the most participants show little to no challenging behavior in the FA, resulting in the inability to identify a behavioral function (25.0%). 

Of the participants who completed the FA and started FCT (*n* = 125), behavioral functions targeted for treatment in FCT continued in a similar pattern to those identified in the FA (Table 4). That is, escape (51.2%) and tangible (60.8%) functions were the most frequently targeted functions for treatment across all project sites.

**Figure 2:**
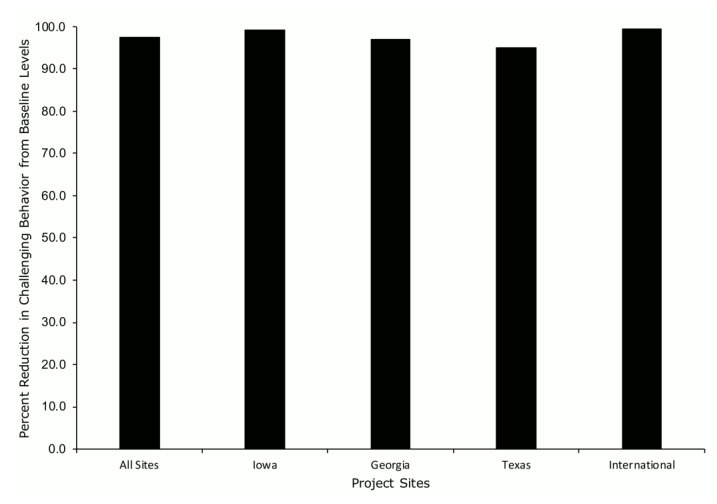
Percent reduction in challenging behavior across project sites.

Of the participants who completed FCT treatment (*n* = 97), the average percent reduction in challenging behavior from baseline levels was 97.4% (SD = 8.2; range, 57–100%; Figure 2). Very similar differences in the percent reduction in challenging behavior occurred across project sites. 

**Figure 3:**
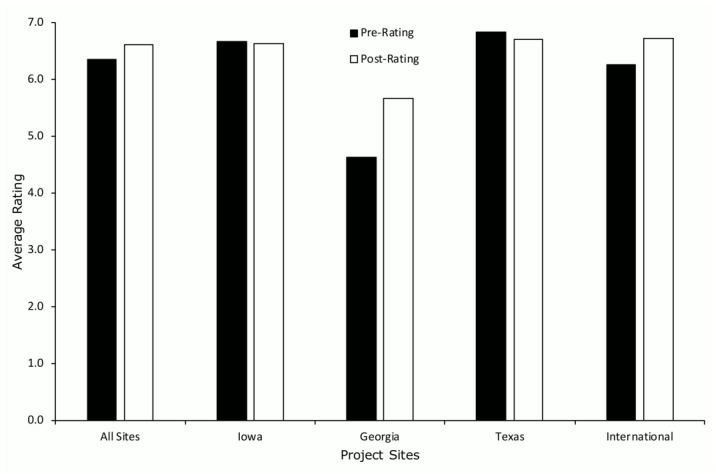
Parent ratings of treatment acceptability using the TARF-R at pre- and post-treatment.

Parent ratings of treatment acceptability averaged 6.4 (SD = 1.0; range 4–7) and 6.6 (SD = 0.8, range 4–7) at pre- and post-treatment, respectively, across all project sites (Figure 3). These results did not differ across sites, except for the generally lower ratings obtained at the Georgia site. Pre-treatment ratings at the Georgia site averaged 4.6 (SD = 1.1, range 4–7) and post-treatment ratings averaged 5.7 (SD = 1.4; range, 4–7). However, variable levels of data were missing across all sites (range, 0% to 72.7%), which may have impacted these results. Specifically, for pre-treatment TARF-R ratings, data were missing for 6.3%, 8.0%, 63.6%, and 66.7% of children at the Texas, International, Georgia, and Iowa sites, respectively. For post-treatment TARF-R ratings, data were missing for 0.0%, 25.0%, 55.6%, and 72.7% of children at the International, Texas, Iowa, and Georgia sites, respectively. 

**Figure 4:**
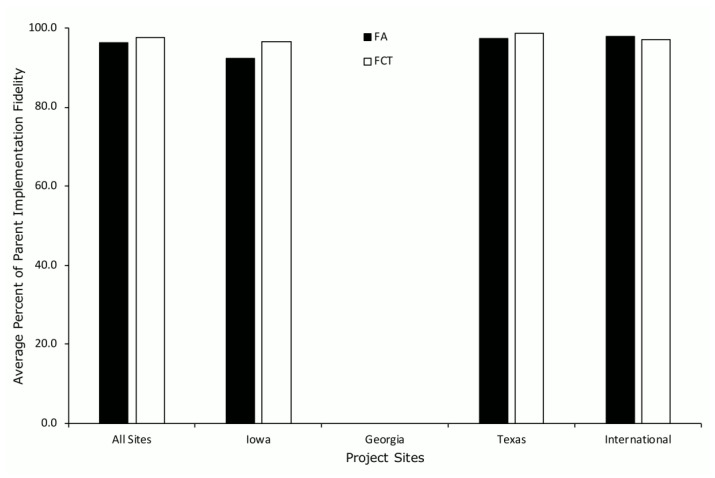
Average percent of parent procedural fidelity during the FA and FCT across project sites.

Parent procedural fidelity during the FA and FCT averaged 96.2% (SD = 6.0; range, 75–100%) and 97.6% (SD = 4.3; range, 70–100%), respectively, across all project sites (Figure 4). Except for the Georgia site, which did not collect parent procedural fidelity data, parent procedural fidelity did not differ across the FA and FCT when separated by project site.

#### 3.2.2. Outcomes for Non-Completers

Of the participants who discontinued participation in the project (*n* = 102), 28.4%, 22.5%, 21.6%, and 27.5% discontinued before the FA, during the FA, before FCT, and during FCT, respectively, across all project sites (Table 5).

In addition to the percent of participants who discontinued participation in the project, Table 5 summarizes the average number of weeks those participants were enrolled in the project prior to discontinuation. For participants who discontinued before the FA was started, the average number of weeks enrolled in the project was 10.2 across all sites. When discontinuation occurred during the FA, the average number of enrolled weeks was 21.9 across all sites. When discontinuation occurred in the FCT phase, the average number of weeks enrolled was 18.3 and 30.2 for those who discontinued before FCT and during FCT, respectively, across all sites.

#### 3.2.3. Outcomes for Completers and Non-Completers

**Figure 5:**
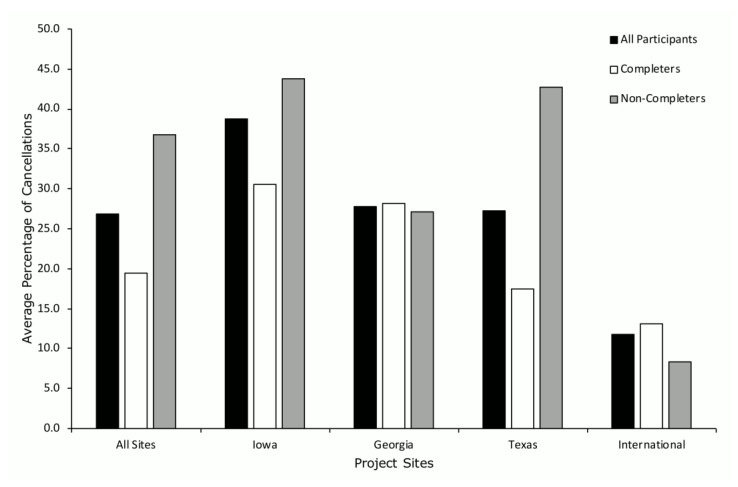
Average percent of visit cancellations across project sites.

Across all participants and all sites (*n* = 199; black bars), the average percentage of visit cancellations was 26.8% (Figure 5) with visit cancellations averaging the lowest for the international site (11.8%) and highest for the Iowa site (38.8%). When cancellations were analyzed by participants who completed the project (*n* = 97; white bars) versus those who discontinued participation in the project (*n* = 102; gray bars), the average percentage of visit cancellations was 19.4% for completers and 36.7% for non-completers across all sites. Similar to the cancellations for all participants by site, the international project showed the lowest average cancellations for completers (13.1%) and non-completers (8.3%), and the Iowa site showed the highest cancellations for completers (30.5%) and non-completers (43.8%).

**Figure 6:**
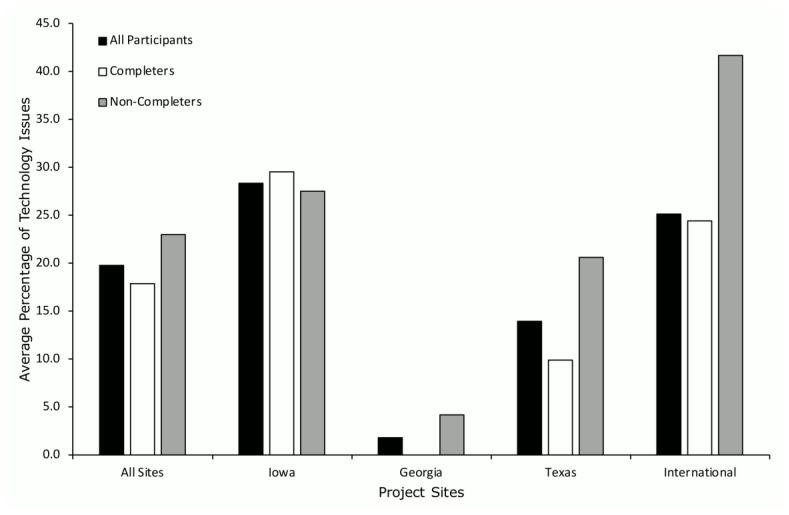
Average percent of technology issues across project sites.

Technology issues (Figure 6) occurred across all sites (black bars) with an average 19.7% of occurrence, with similar occurrences when evaluated by participants who completed the project (17.8%; white bars) and participants who discontinued their participation in the project (22.9%; gray bars). Across all participants, the Iowa and international sites had the highest average occurrence of technology issues (*M* = 28.4% and 25.1%, respectively), and Georgia had the lowest average occurrence of technology issues (*M* = 1.9%). For participants who completed the project, Iowa had the highest occurrence of technology issues (*M* = 29.6%) and Georgia had the lowest occurrence (*M* = 0.0%). For participants who discontinued participation in the project, the international site had the highest occurrence of technology issues (*M* = 41.7%) and Georgia had the lowest occurrence (*M* = 4.2%). However, the Georgia site was also the most inconsistent site for collecting data on cancellations and technology issues.

## 4. Discussion

A feasible and robust service delivery model must be both effective and efficient for providers to adopt, maintain, and expand its use. To date, the use of telehealth as a service delivery model in ABA has most often focused on addressing the challenging behavioral needs of young children with disabilities with largely positive results [17]. More specifically, the FA + FCT model via telehealth, as described by Lindgren et al. [16], was shown to be effective in reducing children’s challenging behavior, efficient in lowering costs and expanding geographical reach, and acceptable to the parents being coached to deliver it. The current study contributes to the evidence of telehealth as a feasible and robust service delivery model when conducting FA + FCT for challenging behavior, especially for young children with ASD. The current study demonstrated the effectiveness and efficiency of FA + FCT via telehealth by showing no systematic differences in reductions in challenging behavior and parent ratings on treatment acceptability across a relatively large sample of national and international participants.

Additionally, the current study demonstrated high levels of parent procedural fidelity in both the FA and FCT across national and international clinical applications, which replicates and extends the study by Suess et al. [23]. Specifically, these results show that parents can reliably implement behavioral assessment and treatment procedures via live remote coaching. As described by Tsami et al. [18], telehealth may provide a unique challenge when interpreters are used because behavioral therapist coaching must be provided in one language and interpreted in a second language before the parent can implement the assessment or treatment procedure. Despite these delays between coaching and implementation, the results of our study showed that parent procedural fidelity with interpreters was maintained at similarly high levels (*M* = 98.6%) to those obtained without the use of interpreters (*M* = 96.9%). These results replicate the findings from Tsami et al. [18], who showed high parent procedural fidelity ranging from an average of 84% to 100% across families who utilized an interpreter versus those who did not. Given these findings, our results provide clinical replication by continuing to demonstrate that parents can implement FA and FCT procedures accurately following coaching from a behavioral therapist, even if implementation is delayed due to the need for coaching to be interpreted.

The combined results from Tsami et al. [18] and the current study indicate that telehealth is an effective and efficient service delivery model for conducting FA + FCT with young children with ASD who display socially maintained challenging behavior. Because of its effectiveness and efficiency, access for families can be expanded beyond local boundaries, which is important given that many families, especially those in rural areas and internationally, have limited or no access to behavioral specialists. For example, 100 active BCBAs are registered in Iowa, whereas no BCBAs are registered in Algeria [28], a country 16 times larger and with 40 million more people than Iowa. Even in Iowa, the majority of BCBAs are located in urban areas within the state, severely limiting access to specialty behavioral services for many families. With the combined results of the present study and previous studies, telehealth provides a path for reducing service access issues both nationally and internationally.

The next logical step is to provide additional clinical replications of this model to be more inclusive of other subgroups of children, settings, and target behaviors. Relative to children, the majority of the telehealth literature has included young children with ASD [17], resulting in the need to expand participant populations to older individuals, as well as those with different developmental diagnoses or none at all. Relative to settings, locations outside of the child’s home have been infrequently studied (e.g., schools, outpatient clinics) [17], and when these settings have been utilized, the behavioral therapist has often been in a secondary location within the building (e.g., [29]), or an assistant has been present in the evaluation room with the family to provide support as needed (e.g., [19,20]). Therefore, further evaluation is warranted to determine the conditions under which telehealth is best utilized in other settings such as group homes, schools, and the community. When including other settings that are likely to involve the presence of additional individuals (e.g., peers, housemates), the consideration of ethical guidelines such as confidentiality and informed consent will be required [30]. Finally, another necessary next step for telehealth is related to target behaviors. As mentioned previously, we did not conduct FAs for challenging behavior that we suspected were automatically maintained because of our uncertainty of how to safely run an alone condition, which requires that the child remain in a room without supervision or access to toys and leisure items. To our knowledge, only two studies have conducted FAs to evaluate automatic reinforcement as maintaining challenging behavior [31,32]. Telehealth poses a potentially unique challenge on how to maintain a child’s safety when challenging behavior occurs in the absence of environmental contingencies. Therefore, future research continues to be needed in this area to determine (a) if telehealth should be used and (b) how it should be used in these cases. Similarly, it is unknown how to utilize telehealth for other challenging behavior such as pica and elopement. Therefore, further study is warranted in these areas to determine the conditions under which telehealth is clinically indicated as a service delivery model.

To further evaluate the feasibility and robustness of the FA + FCT telehealth model, we evaluated variables related to project discontinuation, cancellations, and technology issues. Our review of the ABA telehealth literature suggests that to date, these variables have not been evaluated. Relative to discontinuation in the project, there were no systematic differences in the phase with which families discontinued services across both national and international clinical applications. The reasons for discontinuation were not collected for the US project; therefore, further analysis may be needed. Thus, it is not known if discontinuation rates differ across outpatient, telehealth, and other service delivery models. For the international project, discontinuation reasons included inadequate internet speeds, scheduling conflicts due to time differences or parent work schedules, parent health concerns, family relocation, and the lack of any challenging behavior during sessions. Relative to the lack of challenging behavior during sessions, the lack of any challenging behavior warrants more evaluation, as the lack of occurrence during telehealth sessions may be a substantial clinical concern. As mentioned previously, the lack of challenging behavior occurred at higher levels in the Iowa site than in any other site and occurred at relatively high levels. It is unclear why this result occurred, but one hypothesis we had was that challenging behavior was maintained by idiosyncratic variables that were not evoked by isolated FA contingencies, which were shown to occur within the literature [33]. Thus, future research is needed on how best to identify and evaluate the occurrence of challenging behavior under these idiosyncratic conditions via telehealth. 

Given the variability of the technology and cancellation data across the US and international sites, follow up research on this topic is needed. For the international project, for which more detailed data were collected, cancellations occurred by both the family and behavioral therapist. The reasons for cancellation included illnesses, holidays or vacations, and natural disasters (e.g., flooding, volcano eruptions). Technology issues included poor internet quality or connectivity and electricity blackouts. The frequency of technology issues resulting in a visit cancellation versus continuation was not collected, and this may warrant further evaluation.

Given these preliminary findings on discontinuation, cancellations, and technology issues, future research should systematically evaluate these variables to determine which variables are unique to telehealth service delivery, and thus contribute to barriers to consistent service accessibility. Understanding the variables that contribute to unsuccessful telehealth service delivery should help guide practitioners and researchers in addressing these barriers such that accessing services provided via telehealth is equitable for all interested families. For example, future research should evaluate the frequency with which cancellations and technology issues occur for both the family and the provider to determine if there are systematic differences between who cancels, when and why cancellations occur, and if these outcomes differ when compared to in-person services. Additionally, future research should evaluate the conditions under which families choose not to begin or discontinue telehealth services (e.g., technology-related, preference for in-person services, long waitlists), as well as the relationship between cancellations, no-shows, and service discontinuation.

Specific to telehealth and ABA service delivery, future research should continue evaluating the conditions under which telehealth as a service delivery model is clinically indicated. First, as mentioned previously, the specific target behaviors referred for assessment and treatment need to be considered for their appropriateness for telehealth services. Second, future research should continue to evaluate differences in parent treatment acceptability ratings and the effects of parent procedural fidelity on child behavioral outcomes. Relative to treatment acceptability, in the current study and previous studies [17], these data were not consistently obtained from all participating families. Even though acceptability ratings never fell below a neutral rating, it will be beneficial to consistently collect these data at various treatment time points (e.g., pre-, mid-, and post-treatment) and evaluate their association with the occurrence of children’s challenging behavior and characteristics of the behavioral therapists providing coaching. Such evaluations may further guide our understanding of variables that contribute to the successful and unsuccessful use of telehealth as a service delivery model. As explored by Schieltz et al. [34], evaluations of the correspondence between child behavioral outcomes and parent procedural fidelity outcomes will be important for understanding variables associated with the success of telehealth. For example, consistently poor parent procedural fidelity during telehealth visits may indicate the need for in-person services or supports, at least for a period of time.

Finally, the current study summarized the demographic variables of the children, behavioral therapists, and interpreters, which have been historically underreported within the field of ABA [35]. Because these data can offer opportunities for the broader dissemination and analyses of the behavioral assessment and treatment approaches conducted in ABA, future research should consider the development of a data repository, as well as quantitative analyses of hypotheses that are driven by cultural variables and experience variables, etc.

## 5. Conclusions

The current study extends the growing ABA telehealth literature on the use of FA + FCT to a relatively large sample of national and international clinical applications. Similar to previous studies, these results demonstrate the effectiveness and efficiency of the FA + FCT telehealth model for addressing the challenging behavior needs of young children with ASD by maintaining the outcomes of reduced challenging behavior, high levels of treatment acceptability, and high levels of parent procedural fidelity. Additionally, these results expand the geographical reach of specialty services in the area of challenging behavior to populations around the globe. As more replications of the effectiveness and efficiency of the FA + FCT telehealth model are published, it will be important for barriers contributing to unsuccessful cases to be disseminated to further guide the conditions under which telehealth as a service delivery model is feasible and robust.

## Figures and Tables

**Table 1:** Child participant characteristics across the US and international projects.

Variables	All Sites	All US Sites	Iowa ^1^	Georgia ^2^	Texas ^2^	International ^3^
Participants Enrolled (*n*)	199	152	59	37	56	47
Age (in months)						
Mean (SD)	56.4 (20.6)	51.8 (16.4)	50.6 (15.1)	56.4 (16.8)	49.9 (17.2)	71.5 (25.4)
Range	20–156	20–97	20–78	22–83	20–97	24–156
Sex (% male)	79.9	80.9	81.4	75.7	83.9	76.6
ASD Diagnosis (%)	99.0	100.0	100.0	100.0	100.0	80.4
Race ^4^ (%)						
White		72.4	83.1	59.5	69.6	
Black or African American		13.8	5.1	32.4	10.7	
American Indian/Alaska Native		0.7	1.7	0.0	0.0	
Asian		7.9	3.4	2.7	16.1	
Native Hawaiian/Other Pacific Islander		0.0	0.0	0.0	0.0	
Two or More Races		4.6	6.8	2.7	3.6	
Not Reported		0.7	0.0	2.7	0.0	
Country of Origin ^5^ (%)						
Algeria						2.1
Cameroon						4.3
China						2.1
Costa Rica						2.1
Egypt						2.1
Ghana						2.1
Greece						17.0
India						4.3
Iran						2.1
Mexico						6.4
Morocco						6.4
Nepal						2.1
Nigeria						4.3
Pakistan						12.8
Russia						2.1
Saudi Arabia						6.4
Turkey						6.4
Ukraine						2.1
United Kingdom						2.1
Venezuela						4.3
Vietnam						6.4
Ethnicity (%)						
Hispanic or Latino	19.1	21.1	8.5	5.4	44.6	12.8
One-Way Distance (in km)						
Mean	2484.0	134.5	220.2	114.7	57.5	10078.5
Range	4.8–13,928.9	4.8–1395.3	4.8–1395.3	9.7–1044.5	4.8–346.0	1210.2–13,928.9
Caregiver as Therapist (%)						
Father	6.5	5.9	8.5	0.0	7.1	8.5
Mother	93.0	93.4	91.5	100.0	91.1	91.5
Other	0.5	0.7	0.0	0.0	1.8	0.0
Interpreter Used (%)	13.6	0.0	0.0	0.0	0.0	57.4

^1^ Individual analysis for one child is summarized in O’Brien et al. [26]. ^2^ Summary data for 17 children are included in O’Brien et al. [27]. ^3^ Individual analyses for 13 of the international children are summarized in Tsami et al. [18]. ^4^ Race is reported according to United States census categories for the US project only. ^5^ Country of origin is reported for the international project only.

**Table 2:** Behavior therapist characteristics across the US and international projects.

Variables	All Sites	Iowa	Georgia	Texas	International
Behavior Therapists ^1^ (*n*)	10	6	1	1	3
Age (in years)					
Mean	38.3	40.0	38.0	44.0	35.0
Range	24–53	28–53			24–44
Sex (% male)	20.0	33.3	0.0	0.0	0.0
Race ^2^ (%)					
White	60.0	83.3	100.0	0.0	0.0
Asian	10.0	16.7	0.0	0.0	0.0
Country of Origin ^3^ (%)					
India	10.0	0.0	0.0	0.0	33.3
Greece	10.0	0.0	0.0	100.0	33.3
Turkey	10.0	0.0	0.0	0.0	33.3
Ethnicity (%)					
Hispanic or Latino	0.0	0.0	0.0	0.0	0.0
Highest Education Level (%)					
Doctorate	50.0	66.7	0.0	0.0	33.3
Master’s	30.0	16.7	100.0	100.0	33.3
Bachelor’s	20.0	16.7	0.0	0.0	33.3
Licenses and Certifications ^4^ (%)					
BCBA-D	20.0	33.3	0.0	0.0	0.0
BCBA	30.0	16.7	100.0	100.0	33.3
Licensed Psychologist	40.0	66.7	0.0	0.0	0.0
Experience with ASD (in months)					
Mean	114.0	124.0	228	12	56
Range	12–228	56–196			12–120
Experience in Behavior Analysis (in months)					
Mean	143.2	176.7	180	36	64
Range	24–408	24–408			36–120
Experience with FA and FCT (in months)					
Mean	126.1	169.7	180	24	21
Range	3–396	24–396			3–36
Experience with Telehealth (in months)					
Mean	20.0	30.8	0	0	5.0
Range	0–80	1–80			0–12

^1^ The behavior therapist for Texas also served as one of the behavior therapists for the international project. ^2^ Race is reported according to United States census categories for the US project only. ^3^ Country of origin is reported for the Texas and international sites. ^4^ BCBA-D = board certified behavior analyst-doctoral; BCBA = board certified behavior analyst.

**Table 3:** Interpreter characteristics across the international project.

Variables	International
Interpreters (*n*)	11
Age (in years)	
Mean (SD)	31.6 (6.9)
Range	24–42
Sex (% male)	9.0
Country of Origin (%)	
Cameroon	9.0
China	9.0
Iran	9.0
Mexico	9.0
Nepal	9.0
Pakistan	9.0
Russia	9.0
Saudi Arabia	9.0
United States	9.0
Venezuela	9.0
Vietnam	9.0
Ethnicity (%)	
Hispanic or Latino	18.2
Occupation (%)	
Parent	18.2
Graduate Student	63.6
Practitioner	18.2
Experience in country where family is located (in years)	
Mean (SD)	15.9 (10.0)
Range	0–33
Families Interpreted For (*n*)	
Mean (SD)	2.4 (2.1)
Range	1–8
Families Served in Each Country (%)	
Algeria	4.0
Cameroon	8.0
China	4.0
Costa Rica	4.0
Egypt	4.0
Iran	4.0
Mexico	12.0
Morocco	12.0
Nepal	4.0
Pakistan	4.0
Russia	4.0
Saudi Arabia	12.0
Ukraine	4.0
Venezuela	8.0
Vietnam	12.0
Languages Interpreted for Families (%)	
Arabic	32.0
Farsi	4.0
French	8.0
Mandarin	4.0
Nepalese	4.0
Russian	8.0
Spanish	12.0
Urdu	4.0
Vietnamese	12.0
Experience with FA and FCT (% yes)	9.0
Location of Interpreter (% participants)	
With Behavior Therapist	28.0
With Family	12.0
Other in US	44.0
Other in Family Country	0.0
Other Country	20.0

**Table 4:** Summary of behavioral functions identified in the FA and targeted for treatment in FCT.

Study Phase	All Sites	Iowa	Georgia	Texas	International
Behavioral Function(s) Identified% (*n*)					
Escape	56.5 (83)	52.5 (21)	48.1 (13)	69.6 (32)	50.0 (17)
Tangible	66.7 (98)	47.5 (19)	77.8 (21)	82.6 (38)	58.8 (20)
Attention	25.9 (38)	15.0 (6)	14.8 (4)	50.0 (23)	14.7 (5)
Automatic	0.7 (1)	0.0 (0)	0.0 (0)	2.2 (1)	0.0 (0)
No Function Identified	12.9 (19)	25.0 (10)	11.1 (3)	6.5 (3)	8.8 (3)
Behavioral Function(s) Targeted in FCT% (*n*)					
Escape	51.2 (64)	60.0 (18)	33.3 (8)	57.1 (24)	48.3 (14)
Tangible	60.8 (76)	46.7 (14)	70.8 (17)	66.7 (28)	58.6 (17)
Attention	6.4 (8)	10.0 (3)	4.2 (1)	7.1 (3)	3.4 (1)

**Table 5:** Participant discontinuation from the project and average number of weeks enrolled in the project by study phase and across project sites.

Study Phase	All Sites	Iowa	Georgia	Texas	International
Before FA					
% (*n*)	28.4 (29)	31.7 (13)	20.0 (3)	20.8 (5)	36.4 (8)
Mean Weeks Enrolled	10.2	19.0	0.0	3.0	4.1
During FA					
% (*n*)	22.5 (23)	14.6 (6)	46.7 (7)	20.8 (5)	22.7 (5)
Mean Weeks Enrolled	21.9	62.7	7.9	6.6	7.8
Before FCT					
% (*n*)	21.6 (22)	24.4 (10)	20.0 (3)	16.7 (4)	22.7 (5)
Mean Weeks Enrolled	18.3	28.3	13.7	7.5	9.8
During FCT					
% (*n*)	27.5 (28)	29.3 (12)	13.3 (2)	41.7 (10)	18.2 (4)
Mean Weeks Enrolled	30.2	51.7	10.0	13.2	18.5

## Data Availability

Data analyzed during the current study are available upon reasonable request to the corresponding author.
